# Increasing genomic diversity and evidence of constrained lifestyle evolution due to insertion sequences in *Aeromonas salmonicida*

**DOI:** 10.1186/s12864-016-2381-3

**Published:** 2016-01-12

**Authors:** Antony T. Vincent, Mélanie V. Trudel, Luca Freschi, Vandan Nagar, Cynthia Gagné-Thivierge, Roger C. Levesque, Steve J. Charette

**Affiliations:** Institut de biologie intégrative et des systèmes, Pavillon Charles-Eugène-Marchand, Université Laval, 1030 avenue de la Médecine, Quebec City, G1V 0A6 QC Canada; Centre de recherche de l’Institut universitaire de cardiologie et de pneumologie de Québec (Hôpital Laval), 2725 Chemin Sainte-Foy, Quebec City, G1V 4G5 QC Canada; Département de biochimie, de microbiologie et de bio-informatique, Faculté des sciences et de génie, Université Laval, 1045 avenue de la Médecine, Quebec City, G1V 0A6 QC Canada; Département de microbiologie-infectiologie et immunologie, Faculté de médecine, Université Laval, Quebec City, QC Canada; Food Technology Division, Bhabha Atomic Research Centre, Mumbai, 400085 India

**Keywords:** *Aeromonas salmonicida*, Phylogeny, Mesophilic, Psychrophilic, Insertion sequence

## Abstract

**Background:**

Aeromonads make up a group of Gram-negative bacteria that includes human and fish pathogens. The *Aeromonas salmonicida* species has the peculiarity of including five known subspecies. However, few studies of the genomes of *A. salmonicida* subspecies have been reported to date.

**Results:**

We sequenced the genomes of additional *A. salmonicida* isolates, including three from India, using next-generation sequencing in order to gain a better understanding of the genomic and phylogenetic links between *A. salmonicida* subspecies. Their relative phylogenetic positions were confirmed by a core genome phylogeny based on 1645 gene sequences. The Indian isolates, which formed a sub-group together with *A. salmonicida* subsp. *pectinolytica*, were able to grow at either at 18 °C and 37 °C, unlike the *A. salmonicida* psychrophilic isolates that did not grow at 37 °C. Amino acid frequencies, GC content, tRNA composition, loss and gain of genes during evolution, pseudogenes as well as genes under positive selection and the mobilome were studied to explain this intraspecies dichotomy.

**Conclusion:**

Insertion sequences appeared to be an important driving force that locked the psychrophilic strains into their particular lifestyle in order to conserve their genomic integrity. This observation, based on comparative genomics, is in agreement with previous results showing that insertion sequence mobility induced by heat in *A. salmonicida* subspecies causes genomic plasticity, resulting in a deleterious effect on the virulence of the bacterium. We provide a proof-of-concept that selfish DNAs play a major role in the evolution of bacterial species by modeling genomes.

**Electronic supplementary material:**

The online version of this article (doi:10.1186/s12864-016-2381-3) contains supplementary material, which is available to authorized users.

## Background

The *Aeromonas* genus (also known as aeromonads) has a complex taxonomy, with fourteen species officially recognized in the latest edition of the *Bergey’s Manual of Systematic Bacteriology* [1]. However, recent works like the one of Colston et al. in 2014 showed, based on molecular phylogenies (house-keeping genes and core genomes), average nucleotide identities (ANI), and *in silico* DNA-DNA hybridization (*is*DDH), that aeromonads have a greater taxonomic richness [2].

Aeromonads can be also divided into two groups based on their ability or inability to grow at a mesophilic temperature (37 °C) [3–5]. The best-known representatives of the mesophilic group are *A. hydrophila*, *A. caviae*, and *A. veronii*, all of which are human pathogens [3, 4]. The psychrophilic group (constituted by species that cannot grow at 37 °C) includes *A. salmonicida*, which can be further divided into five subspecies: *salmonicida*, *smithia*, *achromogenes*, and *masoucida* [3] as well as *pectinolytica*, which is an exception to the rule since it grows well at 37 °C and is considered to be mesophilic [6].

*A. salmonicida* is of particular interest since this aeromonad species is known to include fish pathogens causing important economical loss worldwide [7, 8]. The genomes of all the subspecies have been sequenced and have been deposited in public databases such as GenBank or Sequence Read Archive (SRA). The sequenced isolates come from geographical origins as diverse as Argentina, Chile, Canada, the United Kingdom, France, Switzerland, Korea, and Japan. However, among these isolates only the genome of *A. salmonicida* subsp. *salmonicida* A449 was completely sequenced, assembled and annotated [9]. A striking result of this study was the discovery of 10 types of insertion sequences (ISs), which were present in 102 copies (88 complete and 14 partial), compared to the genome of *A. hydrophila*, which contained no ISs [10].

Recently, *A. salmonicida* isolates have also been obtained in India from food samples at a local market [11]. The origin of these *A. salmonicida* isolates (i.e., not directly from water or diseased fish) in a tropical climate is very intriguing in terms of the spread of *A. salmonicida* species. With the exception of the *pectinolytica* subsp., which was isolated from a polluted river in Argentina [6], all the other *A. salmonicida* subsp. were isolated from infected hosts living in cold water [9, 12–17].

We sequenced the genomes and examined the genomic elements of the Indian isolates in order to gain a better understanding of the genome architecture and the wide diversity of *A. salmonicida* species. Consequently, we performed an optimized core-genome phylogeny of the aeromonads including previously sequenced *A. salmonicida* genomes as well as genomes sequenced specifically for the present study. A comparison of these genomes revealed that among aeromonads, *A. salmonicida* subspecies are unusually diverse. It also uncovered at least one new mesophilic subspecies and showed that *A. salmonicida* was evolving from a mesophilic to a psychrophilic lifestyle due to the loss of its ability to proliferate in mesophilic environments. We propose that insertion sequences are an important driving force, which may have locked some *A. salmonicida* subspecies into a psychrophilic lifestyle in order to conserve their genomic integrity.

## Results and discussion

### Sequencing new aeromonad genomes

To shed light on the evolution and diversity of *A. salmonicida* species, we first used next-generation sequencing (NGS) to sequence the complete genomes of five additional isolates, including one member of the subspecies *smithia* (JF4097), one of the subspecies *salmonicida* (RS 534), and three isolates from India with an unclear taxonomy (Y47, Y567, and Y577) [11]. The results of the *de novo* assembly for the five isolates are presented in Additional file 1.

### Phylogenetic analysis of *A. salmonicida*

A robust optimized molecular phylogeny of 43 aeromonads was inferred from 1645 gene sequences (see Additional files 1 and 2) to study the taxonomy of this genus and more specifically the *salmonicida* species. Since our study mainly focused on *A. salmonicida*, we were surprised to see that according to our phylogeny, *A. salmonicida* CBA100, a recently deposited Chilean isolate [12], was phylogenetically closer to *A. bestiarum* than to *A. salmonicida* (see Additional file 1). To verify the relatedness of the CBA100 isolate and *A. bestiarum*, the average nucleotide identity (ANI) values were computed for some key taxa (see Additional file 1). The ANI values were in agreement with the molecular phylogeny since the ANI value of the CBA100 isolate and *A. bestiarum* was above 96 %, meaning that the CBA100 isolate likely belong to the *A. bestiarum* species and not *A. salmonicida* as initially proposed [12]. A recent study based on *in silico* DNA-DNA hybridization (*is*DDH) also suggested that CBA100 isolate is a member of the *A. bestiarum* group [18].

**Fig. 1:**
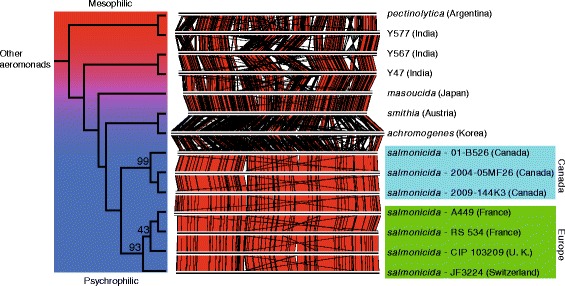
View of the *salmonicida* species section of the phylogenetic tree with proportional branch lengths. See Additional file 1 for the complete tree for all the aeromonads. Putative chromosome sequences were constructed for all the taxa and were compared based on their phylogenetic positions. The red alignments show identity between direct sequences while the gray ones show between inverted sequences. Only the subspecies of each *A. salmonicida* strain are indicated on the right. The geographic provenance of each taxon is indicated in brackets. Only bootstrap values below 100 are shown. The gradient from red to blue represents the ability of each taxon to grow at a mesophilic temperature (red) or at a strict psychrophilic one (blue)

The Indian isolates (Y47, Y567, and Y577) had basal positions among the *salmonicida* species (Fig. 1) and the ANI analysis confirmed that these taxa are *salmonicida* species (see Additional file 1). Isolate Y577 shared a clade with *A. salmonicida* subsp. *pectinolytica* while isolates Y47 and Y567 formed a basal clade to the *masoucida* subspecies. The subspecies *smithia* formed a clade with the subspecies *achromogenes* while the subspecies *salmonicida* RS 534 strain clustered among the other isolates of this subspecies (Fig. 1).

The complete taxon sampling of the *salmonicida* subspecies coupled with the high level of accuracy engendered by the high number of markers (696,249 positions and 420,006 alignment patterns) of our molecular phylogenetic analysis clustered the European and the Canadian isolates independently (Fig. 1). It is interesting to note that the bootstrap value at the node corresponding to the split between the European and the Canadian isolates is 100, which suggests an important statistical robustness. Previous studies using low resolution approaches such as restriction fragment length polymorphism (RFLP) and random amplified polymorphic DNA (RAPD) have indicated that *salmonicida* subspecies strains are genetically homogeneous [19–22] and with a clonal population structure [23, 24]. Another study, based on the sequence of the gene *tapA*, proposed that there was no difference between the European isolates and those found in North America [25]. However, recent studies have shown that isolates from different geographical regions may bear specific variants of the *AsaGEI* genomic island and that *AsaGEI* can be used to track the geographical provenance of *salmonicida* isolates [15, 26]. The present study confirms that there are differences between European and Canadian isolates at the molecular level. On the other hand, the branch lengths were small (see Additional file 1), meaning that the dichotomy between the European and Canadian isolates is a relatively recent event and/or that the European and Canadian isolates have a similar mutation rate. Additional *salmonicida* isolates from other regions need to be analyzed to determine whether they also cluster differently.

### Mesophilic/psychrophilic dichotomy

**Fig. 2:**
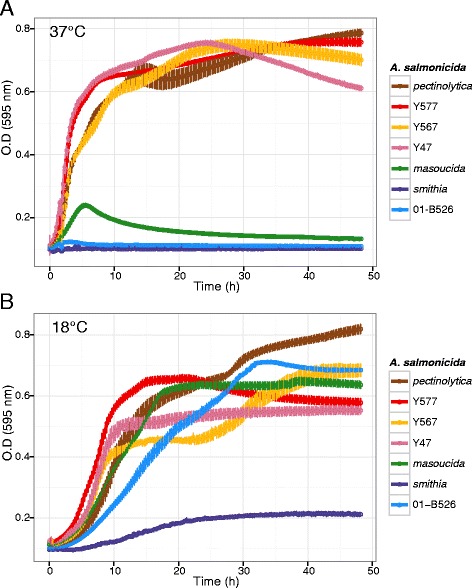
Growth curves at 37 °C (**a**) and 18 °C (**b**) for selected *A. salmonicida* subspecies. The growth curves were determined three times in independent experiments. The means of three replicates with standard error of the means are shown for each subspecies

Since *A. salmonicida* subsp. *pectinolytica* is a mesophilic strain and is closely related to the Indian isolates (Fig. 1), we grew the Indian isolates (Y47, Y567 and Y577) at 18 °C and 37 °C to clarify whether they were mesophilic as well. They grew very well at 37 °C and could thus be considered as mesophilic (Fig. 2a). *A. salmonicida* subsp. *masoucida*, which is considered a psychrophilic subspecies [3], tolerated and even grew at 37 °C albeit slowly and to a lower density before declining. This capacity of *A. salmonicida* subsp. *masoucida* to grow moderately at 37 °C was also reported in a previous large-scale phenotypic study without, however, being clearly mentioned [27]. This is a key observation given that *A. salmonicida* subsp. *masoucida* is positioned in the molecular phylogeny directly after the mesophilic clades and shares the same basal node as the psychrophilic clades (Fig. 1).

The 18 °C temperature has been chosen for additional growth tests since it is at this temperature that psychrophilic *A. salmonicida* subsp. *salmonicida* strains are usually the more efficient to infect fish [28–30]. All the tested isolates, including the mesophiles, grew well at 18 °C (Fig. 2b). Even if 18 °C is the most efficient temperature for *A. salmonicida* to infect fish, we have also tested the growth of these isolates at 7 °C. The growth curves showed the same patterns than at 18 °C, with the mesophilic strains growing more efficiently than the psychrophilic ones (see Additional file 1). Interestingly, the psychrophilic isolates had a lower growth rate capacity than the mesophilic ones at psychrophilic temperatures. This suggests that the psychrophilic isolates did not gain the ability to be psychrophilic but, in fact, experienced an alteration of their overall physiology leading to the loss of their capacity to grow in a mesophilic environment. In addition, the growth profiles of the *pectinolytica* subspecies and Y577 were different, which indicates, in agreement with the overall genomic organization (Fig. 1), that they may belong to different subspecies with a near common ancestor. This is also possible for Y47 and Y567 since they do not present the same growth profile.

Interestingly, the isolate representing the *smithia* subspecies grew minimally at 18 °C and not at all at 37 °C (Fig. 2). According to the *Bergey’s Manual of Systematic Bacteriology* [1], the requirements of this subspecies are not different than those of the other *salmonicida* subspecies. However, we cannot rule out the possibility that the *smithia* subspecies has specific unknown growth requirements.

### Genomic features responsible for the dichotomy

Based on our dataset, it is tempting to suggest that *A. salmonicida* subsp. *masoucida* lies at the interface of a mesophilic/psychrophilic lifestyle. *A. salmonicida* could be an interesting model to study how bacteria gradually evolve from a mesophilic to a psychrophilic lifestyle. We postulate here that, from an evolutionary point of view, *A. salmonicida* is as an example of a recent evolution in lifestyle.

Previous interesting studies have investigated bacteria from different genera to uncover the genomic elements responsible for the mesophilic and psychrophilic lifestyles [31, 32]. However, comparing bacteria from different genera to infer how this type of adaptation has occurred is biased by the noise of unrelated genotypic variations. As reviewed elsewhere [33], bacterial adaptation to different temperatures implies many major physiological changes. Since mesophilic and psychrophilic isolates of *A. salmonicida* are phylogenetically close to each other, genomic variations among these isolates should mostly be related to differences in lifestyle (i.e., mesophilic versus psychrophilic).

Many studies have reported differences in the amino acid compositions of proteins from psychrophilic and mesophilic strains [31, 32, 34, 35]. The ratios of the amino acids of *A. salmonicida* isolates that can grown at 37 °C and those that cannot were computed, and the results were analyzed using an unpaired *t*-test (see Additional file 3). Unlike other studies, we found few significant (*p* < 0.05) differences in amino acid composition (only for Gly, His, and Val) between the two groups. This result was expected given that there was no significant difference between the %G + C values of the two groups (see Additional file 3). However, it is important to note that we were limited by the small number of *salmonicida* subspecies, which resulted in a low statistical power (four mesophilic isolates and three psychrophilic isolates). In addition, it has been already reported that such analyses may give inconsistent results [36].

The total number of tRNA genes (ttRNA) and tRNA diversity (dtRNA) are also correlated with the optimal growth temperature [37], with the psychrophilic bacteria harboring more tRNA genes than the mesophilic ones in order to compensate for a lower diffusion rate. However, despite the low statistical power no significant difference was found between the *A. salmonicida* mesophilic and psychrophilic isolates (see Additional file 3). The fact that there was no significant difference between mesophilic and psychrophilic *A. salmonicida* subspecies in terms of genomic elements that can normally be used to discriminate between lifestyles tends to reinforce the hypothesis that the dichotomy in *A. salmonicida* lifestyles is a recent event and that one lifestyle could be derived from the other (i.e., some strains have lost the ability to grow at 37 °C).

**Fig. 3:**
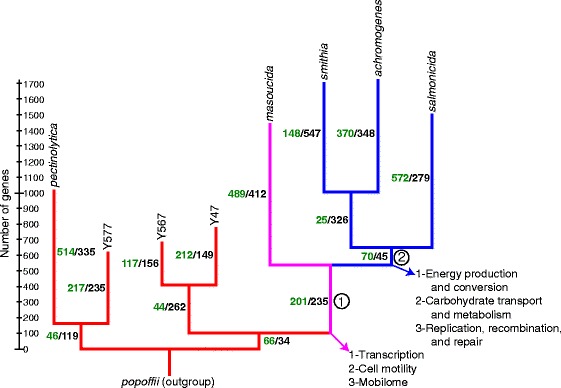
Distribution of the pan-genome on a phylogenetic tree for some key taxa. The phylogenetic tree was based on the tree found using the core genome. The green and black values indicate the number of genes acquired and lost, respectively, for the specific branch using the parsimonious Dollo model. The branch lengths represent the total number of genes acquired or lost. The three functional categories for which genes are the most affected for branches 1 and 2 are indicated. For more details on the analysis of the functional categories see Additional file 1. For *A. salmonicida* subsp. *salmonicida* the strain used was 01-B526

We used an in-house Perl script (see “Methods” section) to find the pan-genome of *A. salmonicida* in order to shed light on the genomic elements responsible for this dichotomy. The resulting binary matrix (i.e., presence/absence) was used to map the characters (i.e., the genes) on a phylogenetic tree based on the core genome (Fig. 3). This analysis made it possible to determine which genes were acquired and which were lost during evolution and, consequently, may have played a role in the adaption of a given isolate. Given the mesophilic-to-psychrophilic gradient, we investigated the gene repertoires for the branch separating *A. salmonicida* subsp. *masoucida* from the mesophilic isolates (branch 1) and the branch separating *A. salmonicida* subsp. *masoucida* from the psychrophilic isolates (branch 2) (Fig. 3).

The functional categories of the genes gained and lost at branches 1 and 2 inferred their potential roles (see Additional file 1). The genes related to branch 1 were present in many functional categories, which made sense given that the mesophilic-to-psychrophilic transition is a complex process and cannot be reduced to a few genes or functional categories. However, it is interesting to note that some categories had acquired or lost many genes. The three most affected categories are transcription (K), cell motility (N), and mobilome (X) (Fig. 3). In the case of branch 2, the three functional categories exhibiting most important changes are energy production and conversion (C) (only losses for this category), carbohydrate transport and metabolism (G), replication, recombination, and repair (L) (Fig. 3). Interestingly only gains have been detected for the category related to the mobilome (X) (see Additional file 1).

The X category (mobilome) appeared to be interesting at both branches. However, since mobile elements are the main cause of contig breaks during the genome assembly process [16, 38], the genes in these categories were likely under-estimated by our analysis. As reviewed elsewhere [39], *A. salmonicida* subsp. *salmonicida* insertion sequences (ISs) are involved in large-scale mutations such as the loss of the type three secretion system (TTSS) [15, 40] (see also Additional file 1), loss of the pAsal1 plasmid [41], the formation of the pAsa6 plasmid from pAsa5 [42], and the disruption of *vapA* [43], a gene encoding the A-layer [44]. More importantly, IS*AS11*, which is involved in the loss of the TTSS locus and the pAsal1 plasmid, is more active when the temperature reaches 25 °C and above [15, 41]. Lastly, the distribution of IS*AS4* (also known as IS*630*) in aeromonads is even enough to construct a clustering of the genus [45], which showed without doubt that *salmonicida* subspecies isolates harbor large numbers of IS*AS4*. Given all of the above, especially knowing that the IS*AS11* is temperature-sensitive, it is tempting to speculate that ISs are involved in the mesophilic-to-psychrophilic transition.

### The involvement of ISs in lifestyle evolution

All the genomes in the present study were in a draft state, with the exception of the *A. salmonicida* subsp. *salmonicida* A449 reference strain [9]. Rigorous study of IS diversity in these genomes is complicated since these mobile elements are one of the main factors behind contig breaks during *de novo* assembly [16, 38, 46]. However, since the drawback is mainly an algorithmic one during the *de novo* assembly process, we decided to address this issue by working directly with sequencing reads. The high sequencing depth provided by Illumina technology allowed us to study IS diversity directly from the raw sequencing data. As indicated in the “Methods” section, the relative abundance of 70 ISs known to be present in *Aeromonas* was computed for the studied members of the *salmonicida* species.

**Fig. 4:**
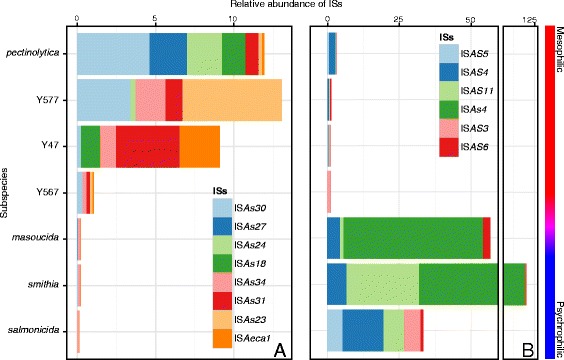
Relative abundance of ISs in *A. salmonicida* subspecies. The distribution of 8 ISs significantly found in the mesophilic isolates (**a**) and 6 in the psychrophilic isolates (**b**) have been determined in a representative set of sequenced *A. salmonicida* isolates

The results of our study of IS diversity showed (1) a dichotomy between mesophilic and the psychrophilic isolates for their IS repertoire and (2) globally a higher number of ISs in the psychrophilic isolates (Fig. 4). In fact, among the 70 ISs studied, 8 were significantly present in the mesophilic isolates and it was possible to see a gradient following the phylogenetic position (Fig. 4a). In the case of the psychrophilic isolates, a striking observation was the high amount of the ISs for the subspecies *masoucida* and *smithia* (Fig. 4b). The ISAs4 was the most abundant IS for both *masoucida* and *smithia*. This IS was previously postulated to be in high copy number in *A. salmonicida* subspecies, at exception of the *salmonicida* subspecies [25]. As indicated previously, IS*AS11*, which causes major rearrangements at high temperatures, was one of the most common ISs in the psychrophilic isolates. For example, the total assembly of the *smithia* subspecies was shown to harbor a very high abundance of IS*AS11*. This observation suggested that the probability of composite transposons is extremely high and, as such, the probability of large-scale genomic rearrangements is also extremely high.

The high copy number of IS*AS11* in the *smithia* subspecies can be partially explained by the presence of the small-high-copy plasmid pJF4097 (see Additional file 1). This plasmid, which is in approximately 40 copies and bears an IS*AS11*, significantly increased the absolute number of IS*AS11* copies. This is a similar situation to that of the *salmonicida* subspecies like 01-B526 in which the pAsal1 plasmid makes an important contribution to the IS*AS11* pool [47]. Of note, both plasmids have different replicons, pJF4097 is a ColE1-type whereas pAsal1 is a ColE2-type replicon. The *smithia* subspecies grew much more slowly at 18 °C than the other psychrophilic isolates (Fig. 2b). It is possible that the metabolic burden caused by the high-copy plasmid and the ISs are responsible for the slow growth of this isolate at 18 °C. In fact, we found that around 14 % of the subsp. *smithia* genome was devoted to ISs. As reviewed recently, ISs have a major impact on genome architecture and evolution and an IS expansion might eventually results in a significant reduction of the genome [48]. This genome reduction might significantly change the bacterial lifestyle, for example by enhancing the transition of a free-living to host-dependent bacteria [48]. It is not impossible that the lifestyle of *A. salmonicida* subsp. *smithia* is currently experiencing dependence to its host, thus explaining the low growth of the bacterium under laboratory conditions.

We believe that it is important to take the absolute number of ISs into consideration since even plasmidic ISs can undergo rearrangements with genomic ISs. For example, a manual investigation of the draft genome of the *A. salmonicida* subsp. *achromogenes* AS03 strain [13] revealed the presence of an IS*AS11* adjacent to the *aopP* gene in a chromosomal contig, just like the pAsal1 plasmid [47].

As recently reviewed [48], ISs not only play significant role in bacterial evolution by shaping the genomic architecture, they can also (1) insert into genes and thus generate pseudogenes and (2) influence expression of neighbor genes. These two genetic impacts are subtler than large-scale deletions or rearrangements, but significantly alter the bacterial behavior, regulation, and lifestyle. Consequently we investigated the pseudogenes annotated in the reference strain A449 [9]. On all 155 known pseudogenes, 21 (13.5 %) were caused by the insertion of an IS. From these 21 pseudogenes, only one could make sense in a context of psychrophilic/mesophilic lifestyle dichotomy: ASA_1469, which was disrupted by an IS*AS4*. This gene encodes a dihydrolipoamide acetyltransferase, a protein member of the pyruvate dehydrogenase complex. This complex has been associated to bacterial lifestyle adaptation [49–51]. An investigation of the sequences of every *A. salmonicida* isolate included in this study showed that all the mesophilic subspecies (*pectinolytica*, Y577, Y567 and Y47) harbor a complete and likely functional gene (i.e. ASA_1469) whereas the psychrophilic subspecies (*smithia*, *achromogenes* and *salmonicida*) and the intermediate one (*masoucida*) have a truncated gene caused by an insertion of IS*AS4* at the same site. This suggests that IS*AS4* was inserted in the gene in the common ancestor of all the psychrophilic (including *masoucida*) subspecies.

Taking altogether, we postulate that ISs play a significant role in the genomic evolution of the *salmonicida* species and that the psychrophilic subspecies may be “locked” into their lifestyle to conserve their genomic integrity. As shown in other studies and confirmed by the present study (see Additional file 1), IS*AS11*s induce major genomic instabilities and prevent bacteria harboring them from growing without inducing genomic rearrangements at 25 °C and above. Moreover, the ISs IS*AS1* and IS*AS2* are also known to be more active around 30 °C [43]. Given all of this, it is reasonable to postulate that other ISs may also induce deletions and rearrangements at higher temperature. In fact, although temperature-sensitive ISs were not frequently documented, there was at least one other case in *Burkholderia multivorans* which was reported [52]. Given the present study, these ISs (i.e., the temperature-sensitive) should be investigated in other psychrophilic bacteria to verify if they can be implied in lifestyle related to growth temperature.

### Genes under positive selection for specific lineages

**Fig. 5:**
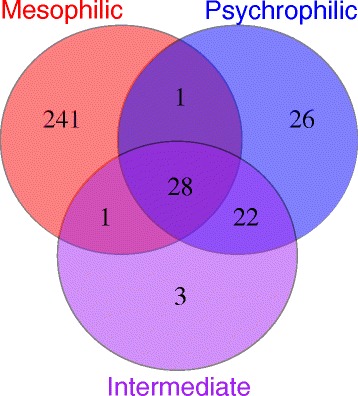
Venn diagram representing the genes under positive selection in the mesophilic, psychrophilic, and intermediate lineages

*A. salmonicida* can be seen as an ideal model for studying relationships between genomic features and bacterial lifestyles given the spectrum of lifestyles of the various subspecies. It would be interesting to determine whether some genes have undergone a positive selection in specific lineages. The core genome of *A. salmonicida*, composed of a balanced dataset of mesophilic and psychrophilic isolates, contained 2758 genes (see Additional file 4), 322 of which were under positive selection in at least one lineage (see the “Methods” section for the details on how the analyses were performed). The categorization of the genes based on lifestyle revealed that those in mesophilic lineages had undergone a more extensive positive selection process than in the psychrophilic lineages (Fig. 5). This leads to at least two hypotheses, which are not mutually exclusive. The first hypothesis is that the mesophilic isolates are able to grow more efficiently than the psychrophilic isolates (Fig. 2) and consequently replicate their genomes more often, making them more prone to accumulating mutations that allow them to respond more quickly to changes in their environment. The second hypothesis is that, since the mesophilic isolates grow well over a wide range of temperatures, many of their genes are subjected to evolutionary pressure to be able to efficiently colonize and adapt to different environments and hosts (which are actually unknown), unlike the psychrophilic isolates, which are subject to less positive selection. However, this does not explain why the psychrophilic isolates evolved from mesophilic isolates.

## Conclusions

Our dataset, which is based on a robust core genome molecular phylogeny, revealed that *A. salmonicida* isolates are much more diverse than previously thought and that they run the gamut of mesophilic, intermediate, and psychrophilic lifestyles. Since mesophilic isolates grow better at 18 °C than psychrophilic isolates, the psychrophilic lifestyle of *A. salmonicida* isolates may be the result of genetic drift rather than adaptation. Our dataset revealed that the ISs may be an important driving and genomic modeling force that pushed and locked the psychrophilic isolates into this lifestyle, much like the transposable elements that have been shown to drive the evolution of *Drosophila melanogaster* [53, 54] and ants [55]. On a more fundamental level, our study provided another important proof-of-concept that selfish DNA can produce evolutionary innovations. However, while it is still unclear why psychrophilic isolates have been conserved throughout the evolution of *A. salmonicida*, there are some possible explanations, including (1) an adaptive selection for psychrophilic hosts like salmonids, (2) a neutral selection, (3) a bottleneck that reduced the effective size of a sub-population of *A. salmonicida*, allowing an enhanced genetic drift and the fixation of the psychrophilic effect in the population, which seems caused by the invasion of ISs, or (4) a relaxation of the selective pressure to grow in mesophilic environments.

## Methods

### Bacterial isolates and growth conditions

The *A. salmonicida* subsp. *smithia* JF4097 isolate was isolated from a diseased Arctic char (*Salvelinus alpinus*) during an ulcerative and hemorrhagic outbreak in Austria [56]. The *A. salmonicida* subsp. *salmonicida* RS 534 isolate, which is also known as A450 [57], was isolated in France. The Indian Y47, Y567, and Y577 isolates were isolated from a chicken and two species of fish (*Ompok bimaculatus* and *Aristichthys nobilis*), respectively, sold as food at a local market in Mumbai [58]. The isolates used in our study were grown on a furunculosis agar [59] at 18 °C for 24 to 72 h. No ethical approval was necessary for any aspect of this study.

### DNA extraction, sequencing, and assembling

The total genomic DNAs of the five isolates were extracted using DNeasy Blood and Tissue kits (Qiagen, Canada). The sequencing libraries were prepared using KAPA Hyper Prep kits and were sequenced by NGS on a MiSeq instrument (Illumina technology) by the Plateforme d'Analyse Génomique of the Institut de Biologie Intégrative et des Systèmes (IBIS, Université Laval). The resulting sequencing reads were assembled *de novo* into contigs using the A5-miseq pipeline version 20140401 [60]. The assemblies were annotated through the NCBI Prokaryotic Genome Annotation Pipeline (PGAP) and deposited under accession numbers [GenBank:JZTF00000000, GenBank:JZTG00000000, GenBank:JZTH00000000, GenBank:JZTI00000000, GenBank:JYFF00000000] for Y47, Y567, Y577, JF4097, and RS 534, respectively. The total genomic DNAs of *A. salmonicida* subsp. *pectinolytica* 34mel^T^ and *A. salmonicida* subsp. *masoucida* NBRC 13784^T^ were also extracted and sequenced as described above. However, the DNA of these isolates were only sequenced to compare ISs, as shown in Fig. 4 (see Results and discussion), and were not *de novo* assembled.

### Phylogenetic analyses

To perform a robust core genome phylogeny, we wrote an in-house Perl script called CoreFinder.pl that relies on BioPerl modules [61] to find the genes involved in the core genome. The script uses coding sequences extracted from a GenBank file and sequentially performs tblastn [62] searches in fasta or multi-fasta (for draft genomes) files. We used *A. hydrophila* ATCC 7966^T^ [10], which is the *A. hydrophila* type strain, as a reference. The genome of this strain has been well studied and has a high-quality annotation. The others aeromonads used in the present study are listed in the Additional file 1.

The coding sequences (CDSs) found by CoreFinder.pl that were involved in the core genomes of the 43 isolates studied (see Additional file 2) were extracted using another in-house Perl script, which uses tblastn in loop. All the gene sequences were aligned using MUSCLE version 3.5 [63]. The resulting alignments were filtered with BMGE version 1.12 [64] to remove constant characters. Lastly, all individual filtered alignments were concatenated into a matrix. This matrix and all the in-house scripts are available upon request to the corresponding author.

The phylogenetic analysis was performed by maximum-likelihood using RAxML version 8.1.17 [65] with the GTR + Γ model and 1000 rapid bootstraps. The best-fit model GTR + Γ was previously chosen by computing the Akaike Information Criterion (AIC) and the Bayesian Information Criterion (BIC) using jModelTest version 2.1.7 [66] (see Additional file 1). The resulting tree was visualized using FigTree version 1.4.2 (http://tree.bio.ed.ac.uk/software/figtree/). To represent the tree, we used a midpoint rooting, an effective method in which the root is defined by the midpoint between the two most divergent operational taxonomic units (OTUs) [67]. The complete optimization steps are available in Additional file 1.

### Assignment of functional categories

An in-house Perl script was written to perform blastp searches to find correspondences between our protein sequences and the Clusters of Orthologous Groups (COGs) database (2014 update) maintained by NCBI [68] in order to assign a functional category to each of them.

### Alignment of the pseudo-chromosomes

To compare the general structure of the chromosome for all isolates of the *salmonicida* species used in this study, we generated pseudo-chromosomes by mapping contigs onto the reference strain A449 of the subspecies *salmonicida* [9], the only chromosome actually fully assembled for the *salmonicida* species, using CONTIGuator version 2.7.4 [69]. The pseudo-chromosome sequences generated were aligned using Easyfig version 2.1 [70] with default parameters.

### Bacterial growth

*A. salmonicida* subsp. *pectinolytica* (34mel^T^), Y577, Y567, Y47, *A. salmonicida* subsp. *smithia* (JF4097), *A. salmonicida* subsp. *masoucida* (NBRC 13784^T^), and *A. salmonicida* subsp. *salmonicida* (01-B526) were inoculated on furunculosis agar [59] or on tryptic soy agar (TSA) from frozen stocks and were grown at 18 °C for 24 to 48 h. The isolates were then inoculated in 2 ml of lysogeny broth (LB) and were incubated at 18 °C overnight. The turbidity was adjusted to an optical density of 0.1 at 595 nm (OD_595_), and the cultures were incubated at 18 °C or 37 °C with shaking at 200 rpm in a Tecan Infinite F200 PRO microplate reader (Tecan, USA). The ODs were read automatically every 15 min for 48 h. The experiments were performed in triplicate.

### Frequency of occurrence of amino acids, %GC, and tRNA composition

The frequency of occurrence of amino acids and the %GC were found for each isolate presented in Additional file 3 using respectively pepstats and geecee included in EMBOSS version 6.6.0.0 [71]. Finally, the tRNA composition (dtRNA and ttRNA) for each isolate was found using tRNA-scan-SE version 1.3.1 [72] by specifying the bacterial search mode. The unpaired t-tests were performed using the statistical framework R [73].

### Pan-genome analyses

An in-house Perl script was used to find the pan-genome of the key taxa (available upon request to the corresponding author). Briefly, the translated coding sequences were extracted from the corresponding GenBank files of each taxon. Since all the genomes had been annotated using PGAP at approximately the same time, there was no bias caused by different annotation processes. The *A. popoffii* genome served as an outgroup. Reciprocal best blast analyses were then performed using blastp. Genes were considered orthologous if their translated sequences shared at least 60 % similarity over at least 85 % of their length. The script encoded the pan-genome as a binary matrix (presence/absence). The genes were considered as Dollo characters in MacClade version 4.08 [74] and were mapped on a phylogenetic tree based on the tree generated using the core genome. This approach has been used in other studies [75, 76].

### Relative IS abundance

The relative abundances of 70 known ISs for the aeromonads listed in ISfinder [77] were determined using the sequencing reads. Briefly, the sequencing reads for each taxon were filtered using Trimmomatic version 0.32 [78] with the parameters suggested in the manual. The resulting filtered sequencing reads were then mapped using BWA (BWA-MEM algorithm) version 0.7.9a-r786 [79] on contigs sequences resulting from a *de novo* assembly (see above for the assembly process) to verify the number of high-quality reads. The reads were then aligned on the 70 IS sequences still using BWA (BWA-MEM algorithm) version 0.7.9a-r786 [79]. The total abundance of each IS was determined by comparing the mapped reads over the total number of high-quality reads and standardized for the IS length using the results of tools included in SAMtools version 0.1.19-44428cd [80].

### Genes under selection

Since we knew the lifestyles and molecular phylogeny of the isolates, we determined whether some genes were under positive selection (dN/dS > 1) for specific lineages. The core genome was found using the same dataset of eight *A. salmonicida* isolates used to find the pan-genome and using the same method used to construct the molecular phylogeny. The well-annotated *A. salmonicida* subsp. *salmonicida* A449 chromosome [9] was used as a reference in this case. All the sequences were codon-aligned using PRANK version 140603 [81]. All the aligned gene sequences were assessed with HyPhy version 2.2.4 [82] using the adaptive branch-site random effects likelihood (aBSREL) method [83]. A gene was considered under positive selection for the tested lineage if the *p*-value (using the Holm-Bonferroni method in HyPhy) was below 0.05.

### Availability of supporting data

The sequence datasets obtained during this project have been deposited in the NCBI GenBank database under the accession numbers [GenBank: JZTF00000000, GenBank: JZTG00000000, GenBank: JZTH00000000, GenBank: JZTI00000000, GenBank: JYFF00000000] for Y47, Y567, Y577, JF4097, and RS 534, respectively. The phylogenetic matrix and the in-house Perl scripts are available upon request to the corresponding author.

## Additional files

Additional file 1:
**Contains additional experimental procedures and results (Table S1.** Aeromonads used in the study; **Table S2.** The five best models and their –InL, AIC, and BIC values; **Table S3.** Assembly results; **Table S4.** Phylogenetic features; Table S5. Biochemical tests used for the mesophilic *A. salmonicida* strains; **Figure S1.** Conceptual schematization of the in-house CoreFinder.pl Perl script; **Figure S2.** Number of genes involved in the core genome based on the similarity percent used with the CoreFinder.pl script; **Figure S3.** Relative abundance of 26 functional categories for genes used to construct the phylogenetic matrixes at 40 and 80 % similarity; **Figure S4.** Molecular core genome phylogeny of 43 aeromonads (80 % similarity); **Figure S5.** Molecular phylogeny of 43 aeromonads (40 % similarity); **Figure S6.** Average nucleotide identity (ANI) analyses for some *A. salmonicida* subspecies included in this study. **Figure S7.** Growth curves at 7 °C for selected *A. salmonicida* subspecies; **Figure S8.** The three high-copy plasmids found in the Indian strain Y47; **Figure S9.** The high-copy plasmid pJF4097 found in *A. salmonicida* subsp. *smithia*; **Figure S10.** Result of the PCR assay confirming that the RS 534 strain lost its TTSS by the recombination of two IS*AS11*s; **Figure S11.** Pan-genome analysis of selected *A. salmonicida* subspecies; **Figure S12.** Functional categories of the genes under positive selection in the *A. salmonicida* mesophilic lineages). (DOCX 3097 kb) Additional file 2:
**List of the coding sequences (CDSs) being in the core genomes of the 43 aeromonad strains studied.** (XLSX 98 kb) Additional file 3:
**The frequency of occurrence of amino acids and the %GC found for each** ***A. salmonicida*** **isolate.** (XLSX 15 kb) Additional file 4:
**List of the coding sequences (CDSs) being in the core genomes of the 8** ***A. salmonicida*** **strains studied.** (XLSX 110 kb)

## Abbreviations

aBSREL: Adaptive branch-site random effects likelihood
ANI: Average nucleotide identities
CDS: Coding sequence
COG: Clusters of orthologous group
IS: Insertion sequence
*is*DDH: *in silico* DNA-DNA hybridization
LB: Lysogeny broth
NGS: Next-generation sequencing
OD: Optical density
OTU: Operational taxonomic unit
PGAP: Prokaryotic genome annotation pipeline
RAPD: Random amplified polymorphic DNA
RFLP: Restriction fragment length polymorphism
TSA: Tryptic soy agar
TTSS: Type three secretion system
