# Supplementary material for: Increasing genomic diversity and evidence of constrained lifestyle evolution due to insertion sequences in Aeromonas salmonicida
Source: BMC Genomics. 2016 Jan 12;17:44. doi: 10.1186/s12864-016-2381-3 (PMC4709979; doi:10.1186/s12864-016-2381-3)
Supplement: Additional file 1: — Contains additional experimental procedures and results (Table S1. Aeromonads used in the study; Table S2. The five best models and their –InL, AIC, and BIC values; Table S3. Assembly results; Table S4. Phylogenetic features; Table S5. Biochemical tests used for the mesophilic A. salmonicida strains; Figure S1. Conceptual schematization of the in-house CoreFinder.pl Perl script; Figure S2. Number of genes involved in the core genome based on the similarity percent used with the CoreFinder.pl script; Figure S3. Relative abundance of 26 functional categories for genes used to construct the phylogenetic matrixes at 40 and 80 % similarity; Figure S4. Molecular core genome phylogeny of 43 aeromonads (80 % similarity); Figure S5. Molecular phylogeny of 43 aeromonads (40 % similarity); Figure S6. Average nucleotide identity (ANI) analyses for some A. salmonicida subspecies included in this study. Figure S7. Growth curves at 7 °C for selected A. salmonicida subspecies; Figure S8. The three high-copy plasmids found in the Indian strain Y47; Figure S9. The high-copy plasmid pJF4097 found in A. salmonicida subsp. smithia; Figure S10. Result of the PCR assay confirming that the RS 534 strain lost its TTSS by the recombination of two ISAS11s; Figure S11. Pan-genome analysis of selected A. salmonicida subspecies; Figure S12. Functional categories of the genes under positive selection in the A. salmonicida mesophilic lineages). (DOCX 3097 kb) [file 12864_2016_2381_MOESM1_ESM.docx]

**SUPPLEMENTARY INFORMATION**

**Increasing genomic diversity and evidence of constrained lifestyle evolution due to insertion sequences in *Aeromonas salmonicida***

Antony T. Vincent^a,b,c^, Mélanie V. Trudel^a,b,c^, Luca Freschi^a,e^, Vandan Nagar^d^, Cynthia Gagné-Thivierge ^a,b,c^, Roger C. Levesque^a,e^, Steve J. Charette^a,b,c#^

a. Institut de biologie intégrative et des systèmes, Pavillon Charles-Eugène-Marchand, Université Laval, 1030 avenue de la Médecine, Quebec City, QC, Canada, G1V 0A6

b. Centre de recherche de l’Institut universitaire de cardiologie et de pneumologie de Québec (Hôpital Laval), 2725 Chemin Sainte-Foy, Quebec City, QC, Canada, G1V 4G5

c. Département de biochimie, de microbiologie et de bio-informatique, Faculté des sciences et de génie, Université Laval, 1045 avenue de la Médecine, Quebec City, QC, Canada G1V 0A6

d. Food Technology Division, Bhabha Atomic Research Centre, Mumbai, 400085, India

e. Département de microbiologie-infectiologie et immunologie, Faculté de médecine, Université Laval, Quebec City, QC, Canada

^#^ Corresponding author: Steve J. Charette, Institut de Biologie Intégrative et des Systèmes (IBIS), Pavillon Charles-Eugène-Marchand, 1030 avenue de la Médecine, Université Laval, Quebec City, QC, Canada G1V 0A6

Telephone: 418-656-2131, ext. 6914, Fax: 418-656-7176

[steve.charette@bcm.ulaval.ca](mailto:steve.charette@bcm.ulaval.ca)

**Supplementary Experimental Procedures**

Phylogenetic analyses

To perform a robust core genome phylogeny, we wrote an in-house Perl script called CoreFinder.pl that relies on BioPerl modules [1] to find the genes involved in the core genome. The script uses coding sequences extracted from a GenBank file and sequentially performs tblastn [2] searches in fasta or multi-fasta (for draft genomes) files (Figure S1). We used *A. hydrophila* ATCC 7966^T^ [3], which is the *A. hydrophila* type strain, as a reference. The genome of this strain has been well studied and has a high-quality annotation. The others aeromonads used in the present study are listed in Table S1. The parameter used to search the CDSs was at least a 85% query cover for various similarity percent (25% to 100%, with 5% steps). The graphical interpretation of the results revealed three linear sections and two breakpoints estimated at 40% and 80% similarity (Figure S2). To verify the importance of this parameter (e.g., the similarity percent) with respect to the final phylogeny, we performed all subsequent analyses (as indicated in the main manuscript) at 40% and 80% similarity.

To choose the most appropriate phylogenetic model, the Akaike Information Criterion (AIC) and the Bayesian Information Criterion (BIC) were computed using jModelTest version 2.1.7 [4] for both matrixes. In both cases, while the best-fit model was GTR+Γ closely followed by GTR+I+Γ (Table S2), there was no significant difference between the two. However, as discussed and reviewed elsewhere [5], the consideration of a rate class with a rate zero caused by invariable sites is meaningless since the α parameter, which governs the shape of the gamma distribution, already allows low-rate sites through an L-shaped gamma distribution caused by an α < 1. Moreover, the use of the mixture model +I+Γ might result in an over-parameterization since it would be difficult to optimize both parameters. We thus used the GTR+Γ model for both matrixes at 40% and 80% similarity.

Species relatedness was inferred by average nucleotide identity (ANI) analyses for key taxa using JSpecies version 1.2.1 [6]. MUMmer version 3.23 [7] was used to perform the analyses since it provides more robust results for genomes sharing a high level of similarity (ANI > 90%) than blast searches [6]. Two taxa were considered to be members of the same species if they shared an ANI ≥ 96%, a value that is well adapted to the aeromonads [8].

Bacterial growth at 7°C

The Indian isolates (Y577, Y567 and Y47) as well as *A. salmonicida* subsp. *pectinolytica* (34mel^T^), *A. salmonicida* subsp. *smithia* (JF4097), *A. salmonicida* subsp. *masoucida* (NBRC 13784T), and *A. salmonicida* subsp. *salmonicida* (01-B526) were inoculated on furunculosis agar or on tryptic soy agar (TSA) from frozen stocks and were grown at 18°C for 24 to 48 h. The isolates were then inoculated in 3 ml of lysogeny broth (LB) and were incubated at 7°C overnight with shaking at 200 rpm. The turbidity was adjusted to an optical density of 0.1 at 595 nm (OD595), and the cultures were incubated at 7°C with shaking at 200 rpm. The ODs were read systematically every hour for 8 h. The experiment was performed in triplicate.

PCR assays

We performed PCR assays using previously published conditions [9] to verify whether the pAsa5 plasmid of strain RS 534 had lost its type three secretion system (TTSS) by the recombination of IS*AS11*B and IS*AS11*C [10].

Plasmid characterization

The contigs for the strains sequenced in the present study were locally mapped on the chromosome sequence of the *A. salmonicida* reference strain A449 [11], the only *A. salmonicida* strain with a fully assembled chromosome, using CONTIGuator version 2.7.4 [12]. Identity searches of the unmapped contig sequences were performed by blast searches against the NCBI nr/nt database. Sequence manipulations were performed using the bioinformatics package EMBOSS version 6.6.0.0 [13].

The plasmid sequences that were discovered were automatically annotated by the RAST webserver [14]. All the putative CDSs were manually curated by performing blastp searches against the NCBI nr/nt database. Putative toxin-antitoxin systems were found by TAfinder [15].

The average copy number of each new plasmid for each chromosome was calculated using the sequencing depth, a procedure that has been successfully used in the past [16]. We filtered the sequencing reads using Trimmomatic version 0.32 [17] with the parameters suggested in the manual. The resulting filtered sequencing reads were mapped on the *gyrB* gene (single copy per chromosome) with CUSHAW3 version 3.0.3 [18] without allowing any mismatches in order to avoid cross-mapping from reads related to other genes. The reads were also mapped on the plasmid sequences. The average coverages were calculated using Qualimap version 2.1.1-dev [19].

Biochemical tests

Three Indian *A. salmonicida* isolates (Y47, Y567 and Y577) were further phenotypically characterized using a set of biochemical tests as described by Pavan *et al*. [20] and Abbott *et al*. [21]. All tests were performed in triplicate and according to conventional protocols with suitable positive and negative controls and incubated at 35°C for 48 – 72 h (unless mentioned). The tests for carbohydrate fermentation and extracellular enzymes were read daily for 7 days; whereas, tests for Voges-Proskauer, polypectate degradation and production of brown diffusible pigment on tryptic soya agar (TSA) were incubated at 25°C for 2 to 4 days.

**Supplementary results**

Sequencing results

Despite the stable average coverage of the assemblies, the N50 values, which are an indicator of contigs length, varied considerably (Table S3). For example, the Y567 Indian strain had a N50 value two times higher than the other strains while JF4097 (*smithia*) had a lower N50 value and the smallest large contig. Large repeated elements such as ISs, duplicated genes, and ribosomal RNA clusters cause contig breaks during *de novo* assembly [22], which suggests that the *A. salmonicida* subsp. *smithia* genome contained numerous large repeated elements.

Molecular phylogeny optimization

At 40% similarity (of the translated sequences), the core genome was estimated at 1645 genes compared to 1190 genes at 80%. The functional categories of the genes (at 40 and 80%) were found using an in-house Perl script as explained in the main manuscript to verify whether there was an enrichment of one or more categories. There were major differences in the relative abundance of the functional categories at 40% and 80% similarity in only two categories (J: translational, ribosomal structure and biogenesis and K: transcription), indicating that the gains/losses were uniform in the other categories (Figure S3). The relative importance of the J category at 80% is higher than at 40%, which is consistent with this conserved process. The high relative importance for the K category at 40% is in accordance with the capacity of various aeromonads to react to a wide diversity of stimuli.

The basic features of the phylogenetic analyses are presented in the Table S4. The matrix at 80% similarity had 35% fewer sites than the matrix at 40% similarity. In both cases, the values of the alignment patterns, which are the numbers of different patterns in the matrixes, corresponded to approximately 60% of the total number of sites. There was no significant difference between the α parameters of the two phylogenetic analyses as estimated by RAxML, meaning that the 35% more sites at 40% similarity shared the same rate as the other sites.

There were differences between the resulting trees in terms of bootstrap values and topology (Figures S4 and S5). In fact, the phylogenetic analysis at 80% similarity had the weakest bootstrap values (Figure S4), indicating that the 35% more sites obtained at 40% similarity are important for obtaining a more robust tree (Figure S5). The topology diverged for the clade containing *Aeromonas veronii*. This observation was understandable since this clade had a weak bootstrap value, even with the matrix at 40% similarity (Figure S5). Based on the bootstrap values, we thus believe that the tree based on the core genome found at 40% similarity more accurately represents the true evolution links between the taxa, which is why we used this phylogenetic tree for the remainder of the study.

Phylogenetic position of *A. salmonicida*

As mentioned in the main manuscript, the molecular phylogeny of the present paper revealed that *A. salmonicida* CBA100, a recently deposited Chilean strain [23], is phylogenetically closer to *A. bestiarum* than to *A. salmonicida* (Figure S5). To verify the relatedness of the CBA100 strain and *A. bestiarum*, the average nucleotide identity (ANI) values were computed for some key taxa (Figure S6). The fact that the ANI value between CBA100 and *A. bestiarum* is above 96% reinforce the close evolutionary link between both taxa and let believe at a miss-classification of CBA100.

Strain Y577 shared a clade with *A. salmonicida* subsp. *pectinolytica*. Based strictly on the molecular phylogeny and the ANI values, we cannot rule out the possibility that Y577 is in fact *A. salmonicida* subsp. *pectinolytica*. As previously published, the *pectinolytica* subspecies is the only known aeromonad with pectinase activity [20]. Interestingly, all the genes in the *pectinolytica* subspecies that are needed to degrade and use pectin as a carbon source [24] were found in the genome of Y577. Given this, the pectinase activity of Y577 was verified and was confirmed experimentally (Table S5). It is tempting to suggest that Y577 is a member of the *pectinolytica* subspecies or a new subspecies sharing a near common ancestor. However, the overall chromosomal organizations of Y577 and the *pectinolytica* subspecies strains appeared divergent (Figure 1, main manuscript), which is unusual for such closely related strains given the chromosomal uniformity of *salmonicida* subspecies strains. The results of some other biochemical tests also diverged (Table S5), suggesting that strains Y577 and *A. salmonicida* subsp. *pectinolytica* 34mel^T^ may not belong to the same subspecies.

Strains Y47 and Y567 formed a basal clade to the *masoucida* and *salmonicida* subspecies. However, like the relation between Y577 and the *pectinolytica* 34mel^T^ strain, we cannot infer that Y47 and Y567 belong to the same subspecies based solely on the molecular phylogeny and the ANI values, especially since there were also macro-chromosomal differences between the two strains. If they belong to the same subspecies, this would indicate that they display significant genomic plasticity. There are also differences between many of the biochemical test results (Table S5), which also points to a potential taxonomic difference. Surprisingly, Y47 and Y567 were also pectinolytic. While 34mel^T^ and Y577 bore genes coding for three lyases involved in the first step of pectin degradation, Y567 and Y47 did not. The genomes of strains Y567, Y47, and Y577 (as a positive control) were annotated using the RAST webserver [25] to verify whether they possessed a subsystem related to pectin degradation. The annotation of Y577 contained the three lyases (EC 4.2.2.2, EC 4.2.2.6 and EC 4.2.2.9) in the “D-galacturonate and D-glucuronate utilization” subsystem while the annotations of Y47 and Y567 did not contain any enzymes involved in pectin degradation. The pectinase activities of Y47 and Y567 likely involved an unknown pathway and are potentially the result of convergent evolution (i.e., when compared to the strains *pectinolytica* 34mel^T^ and Y577). This result is interesting since it evokes that pectinolytic activity could be important for mesophilic *A. salmonicida*.

Bacterial growth at 7°C

The capacity of various *A. salmonicida* isolates to grow at 7°C was tested in addition to 18°C and 37°C (main manuscript). The same trend as at 18°C was observed, with the mesophilic strains growing more efficiently than the psychrophilic ones (Figure S7). The isolate JF4097 of the subspecies *smithia* was not able to grow at this temperature. This was expected knowing that this isolate had a weak growth capacity at 18°C (main manuscript).

Investigation of the plasmidome

The putative chromosomal contigs were removed and the remaining contigs were analyzed in order to investigate the plasmidome of the strains for which the DNA was sequenced in the present study. This resulted in the identification of three small cryptic plasmids in Indian strain Y47 (Figure S8). To our knowledge, this was the first time that these plasmids had been found. We named them pY47-1, pY47-2, and pY47-3 and deposited their sequences in GenBank under accession numbers KT334396, KT334397, and KT334398, respectively. There were no clear known functions associated with these plasmids. All bore a putative type II toxin-antitoxin maintenance system and/or a phage resistance mechanism [26]. The plasmids pY47-2 and pY47-3 are ColE2-type replicon plasmids with a short RNA (RNA I) replication regulator [27]. Interestingly, a blastn (word size of 11) search revealed sequence identity and structural similarity between pY47-3 and the ColE2-type replicon plasmids pAQ2-1 and pAQ2-2 in *Aeromonas sobria* and *Aeromonas hydrophila*, respectively [28]. However, unlike these plasmids, pY47-3 did not bear the *qnrS2* quinolone resistance gene.

The high sequencing depth provided by the Illumina technology was used to infer the average copy number per chromosome of each plasmid. As it has been reported elsewhere [29], high copy numbers of ColE2-type replicon plasmids are maintained per cell (~ 24 copies for pY47-2 and ~13 copies for pY47-3). The plasmid pY47-1, for which the incompatibility group is unknown, also had a high copy number (~22 copies). It is important to mention that inferring relative plasmid copy numbers has an inherent bias since it is assumed that there is a single copy of the chromosome in each cell, which is not true after the replication. However, the results showed that these plasmids were maintained at much higher copy numbers than the bacterial chromosome. No plasmids were found in strain Y567 while Y577 harbored a pY47-3 plasmid that shared more than 99% identity with the one in Y47 (7 point mutations).

A plasmid maintained at a high copy number (~40 copies/cell) was also found in *A. salmonicida* subsp. *smithia* JF4097 and was subsequently named pJF4097 and its sequence deposited in GenBank under the accession number: KT334395 (Figure S9). pJF4097 bears the *mobABCD* genes, which are related to mobilization, an IS*AS11*, and a gene encoding an ExoY-like protein, which is a type-three secretion system (TTSS) effector in the human pathogen *Pseudomonas aeruginosa* [30].

The *A. salmonicida* subsp. *salmonicida* RS 534 strain harbors the same five plasmids as the A449 reference strain [11], including the large plasmid pAsa4, which encodes many drug resistance genes; pAsa5, which normally bears the type-three secretion system, and the pAsa1, pAsa2, and pAsa3 cryptic plasmids [31]. Basic bioinformatics analyses showed that the pAsa5 plasmid of the RS 534 strain has lost its TTSS. It is known that this region is bordered by two IS*AS11*s (B and C) and that growth above 25°C may result in the recombination of the two IS*AS11*s and the loss of the TTSS [9,10]. We confirmed by PCR that the TTSS was lost by a recombination of IS*AS11*B and C (Figure S10).

The pan-genome analyze

We used an in-house Perl script as indicated in the main manuscript to find the pan-genome of *A. salmonicida*. The resulting binary matrix (i.e., presence/absence) was used to map the characters (i.e., the genes) on a phylogenetic tree based on the core genome (Figure S11A). This analysis made it possible to determine which genes were acquired or lost during evolution and, consequently, may have played a role in the adaption of a given strain. As indicated in the main manuscript, three functional categories (K, N and X) at branch 1 experienced many events (i.e., gains and losses) (Figure S11B). The L, R, T and U categories have also acquired and lost many genes, but this can more likely be attributed to general rather than mesophilic-to-psychrophilic evolution. In the case of branch 2 (Figure S11C), the three functional categories exhibiting most important changes are energy production and conversion (C) (only losses for this category), carbohydrate transport and metabolism (G), replication, recombination, and repair (L). Interestingly only gains have been detected for the category related to the mobilome (X).

Unfortunately, it was impossible to assign a cluster of orthologous groups (COGs) at 45,4 and 59,1% of the genes for the branches 1 and 2, respectively and, consequently, to infer their functional categories. This highlights a drawback of bioinformatics analyses and their dependence on incomplete and poorly curated databases.

Functional categories of the genes under positive selection in the mesophilic lineages

A total of 322 genes in the *A. salmonicida* lineages appear to be under positive selection for various lineages among the *salmonicida* species, including 241 that were specific to at least one mesophilic lineage. We used a COG assignment of these 241 genes to find their relative functional categories (Figure S12). Many categories in the mesophilic lineages were under positive selection, indicating that these lineages may have a high evolutionary potential.

**Table S1.** Aeromonads used in the study.

| **Species** | **Strain** | **Accession no.** | **Reference** |
| --- | --- | --- | --- |
| *A. allosaccharophila* | CECT 4199^T^ | CDBR00000000 | [8] |
| *A. allosaccharophila* | BVH88 | CDCB00000000 | [8] |
| *A. australiensis* | CECT 8023^T^ | CDDH00000000 | [8] |
| *A. bestiarum* | CECT 4227^T^ | CDDA00000000 | [8] |
| *A. bivalvium* | CECT 7113^T^ | CDBT00000000 | [8] |
| *A. caviae* | CECT 838^T^ | CDBK00000000 | [8] |
| *A. dhakensis* | CIP 107500 | CDBH00000000 | [8] |
| *A. diversa* | CECT 4254^T^ | CDCE00000000 | [8] |
| *A. encheleia* | CECT 4342^T^ | CDDI00000000 | [8] |
| *A. enteropelogenes* | CECT 4487^T^ | CDCG00000000 | [8] |
| *A. eucrenophila* | CECT 4224^T^ | CDDF00000000 | [8] |
| *A. fluvialis* | LMG 24681^T^ | CDBO00000000 | [8] |
| *A. hydrophila*^a^ | ATCC 7966^T^ | CP000462 | [3] |
| *A. jandaei* | CECT 4228^T^ | CDBV00000000 | [8] |
| *A. media* | CECT 4232^T^ | CDBZ00000000 | [8] |
| *A. molluscorum* | 848^T^ | AQGQ00000000 | [32] |
| *A. piscicola* | LMG 24783^T^ | CDBL00000000 | [8] |
| *A. popoffii* | CIP 105493^T^ | CDBI00000000 | [8] |
| *A. rivuli* | DSM 22539^T^ | CDBJ00000000 | [8] |
| *A. salmonicida* subsp*. salmonicida* | A449 | CP000644 | [11] |
| *A. salmonicida* subsp*. salmonicida* | 01-B526 | AGVO01000000 | [33] |
| *A. salmonicida* subsp*. salmonicida* | RS534 | JYFF00000000 | This study |
| *A. salmonicida* subsp*. salmonicida* | JF3224 | JXTA00000000 | [9] |
| *A. salmonicida* subsp*. salmonicida* | CIP 103209 | CDDW00000000 | [8] |
| *A. salmonicida* subsp*. salmonicida* | 2009-144K3 | JRYV00000000 | [34] |
| *A. salmonicida* subsp*. salmonicida* | 2004-05MF26 | JRYW00000000 | [34] |
| *A. salmonicida* | CBA100 | JPWL00000000 | [23] |
| *A. salmonicida* subsp*. achromogenes* | AS03 | AMQG00000000 | [35] |
| *A. salmonicida* subsp*. smithia* | JF4097 | JZTI00000000 | This study |
| *A. salmonicida* subsp*. pectinolytica* | 34mel^T^ | ARYZ00000000 | [36] |
| *A. salmonicida* subsp*. masoucida* | NBRC 13784^T^ | BAWQ00000000 | N/A^b^ |
| *A. salmonicida* | Y47 | JZTF00000000 | This study |
| *A. salmonicida* | Y567 | JZTG00000000 | This study |
| *A. salmonicida* | Y577 | JZTH00000000 | This study |
| *A. sanarellii* | LMG 24682^T^ | CDBN00000000 | [8] |
| *A. schubertii* | CECT 4240^T^ | CDDB00000000 | [8] |
| *A. simiae* | CIP 107798^T^ | CDBY00000000 | [8] |
| *A. sobria* | CECT 4245^T^ | CDBW00000000 | [8] |
| *A. species* | AH4 | ERX552948^c^ | [8] |
| *A. species* | AMC34 | AGWU00000000 | N/A |
| *A. taiwanensis* | LMG 24683^T^ | CDDD00000000 | [8] |
| *A. tecta* | CECT 7082^T^ | CDCA00000000 | [8] |
| *A. veronii* | CECT 4257^T^ | CDDK00000000 | [8] |

a: This strain was used as a model to find the genes involved in the core genome.

b: N/A means that no publication is associated with the sequence.

c: Only the sequencing reads were available via the SRA database for *A*. species AH4. The reads were *de novo* assembled as indicated in the “Methods of the main manuscript” section.

**Table S2.** The five best models and their –InL, AIC, and BIC values.

|  | **Similarity** | | | | | |
| --- | --- | --- | --- | --- | --- | --- |
|  | **40%** | | | **80%** | | |
| **Model** | **-lnL** | **AIC** | **BIC** | **-lnL** | **AIC** | **BIC** |
| GTR+Γ | 12827871 | 25655928 | 25656993 | 7941102 | 15882391 | 15883417 |
| GTR+I+Γ | 12827940 | 25656069 | 25657146 | 7941148 | 15882484 | 15883521 |
| HKY+Γ | 12832779 | 25665737 | 25666756 | 7944260 | 15888698 | 15889679 |
| HKY+I+Γ | 12832849 | 25665878 | 25666909 | 7944305 | 15888791 | 15889783 |
| SYM+Γ | 13005302 | 26010784 | 26011815 | 8071711 | 16143602 | 16144595 |

**Table S3.** Assembly results.

|  | **Strains** | | | | |
| --- | --- | --- | --- | --- | --- |
|  | **Y47** | **Y567** | **Y577** | **JF4097** | **RS 534** |
| **Contigs** | 118 | 47 | 104 | 344 | 123 |
| **Largest contigs (kbp)** | 395.27 | 448.10 | 383.04 | 109.61 | 382.43 |
| **N50 (kbp)** | 117.77 | 217.34 | 101.76 | 28.95 | 119.02 |
| **Average coverage** | 66.92 | 68.03 | 78.53 | 88.72 | 62.59 |
| **Assembly size (Mbp)** | 4,710233 | 4,554847 | 4,736410 | 4,307768 | 4,889640 |
| **A449 fraction (%)^a^** | 85.077 | 85.607 | 83.731 | 84.059 | 97.599 |

a: The chromosome sequence of the strain A449 (*A. salmonicida* subsp. *salmonicida*) [11] was used. This feature was found using QUAST version 3.1 [37].

**Table S4.** Phylogenetic features.

|  | **Similarity percent** | |
| --- | --- | --- |
|  | **40%** | **80%** |
| **Genes** | 1645 | 1190 |
| **Sites** | 696,249 | 454,574 |
| **Alignment patterns** | 420,006 | 271,519 |
| **Best model** | GTR+Γ | GTR+Γ |
| **α parameter** | 1.703415 | 1.686099 |

**Table S5.** Biochemical tests used for the mesophilic *A. salmonicida* strains.

| **Biochemical tests** | **Strains** | | | |
| --- | --- | --- | --- | --- |
|  | **34mel^T a^** | **Y577** | **Y47** | **Y567** |
| Indole (35°C) | + | + | + | + |
| ONPG | + | + | + | + |
| VP (25°C) | + | + | + | + |
| Simmons citrate | + | + | + | + |
| Esculin hydrolysis | - | + | - | + |
| Polypectate degradation (25°C) | + | + | + | + |
| Motility (35°C) | - | + | + | + |
| Brown pigment (25°C) | + | - | - | - |
| Growth (37°C) | + | + | + | + |
| Dnase | + | + | + | + |
| Lipase | + | + | + | + |
| Gelatinase | + | + | + | + |
| H_2_S | - | - | - | - |
| VP (35°C) | - | - | - | - |
| ODC | - | - | - | - |
| LDC | - | + | + | + |
| ADH | - | + | + | + |
| Urease | - | - | - | - |
| Cellobiose | + | + | - | + |
| Salicin | - | + | - | + |
| Sorbitol | + | + | + | + |
| Rhamnose | - | - | - | - |
| Mannitol | + | + | + | + |
| Sucrose | + | + | + | + |
| Glucose (gas) | + | + | + | + |
| L-Arabinose | + | + | + | + |
| Lactose | + | + | + | + |
| Glycose | + | + | + | + |
| Inositol | - | - | - | - |
| Melibiose | - | - | - | - |
| Glu | + | + | + | + |
| Amygdalin | - | + | - | + |
| Hemolysin (sheep, horse)^a^ | + | + | + | + |

a: These results are from [20]. We have used horse blood agar for assessing hemolysis; whereas, Pavan et al. (2000) [20] have used sheep blood agar plates.

**Figure S1.** Conceptual schematization of the in-house CoreFinder.pl Perl script.

**Figure S2.** Number of genes involved in the core genome based on the similarity percent used with the CoreFinder.pl script. The blue dots at 40% and 80% indicate the similarity percent used to perform the optimization analyses.

**Figure S3.** Relative abundance of 26 functional categories for genes used to construct the phylogenetic matrixes at 40 and 80% similarity.

**Figure S4.** (A) Molecular core genome phylogeny of 43 aeromonads inferred from the sequences of 1190 genes (determined using the 80% similarity) by maximum-likelihood using the GTR+Γ model and a 1000 rapid bootstrap analysis. Only bootstrap values under 100 are shown. For clarity, the bootstrap values have been removed for the taxa of the *salmonicida* species. The mesophilic strains are in red while the psychrophilic strains are in blue. (B) Zoom of *salmonicida* species with equal branch lengths. Only bootstrap values under 100 are shown. The mesophilic, intermediate, and psychrophilic strains are shown in red, purple, and blue, respectively.

**Figure S5.** Molecular phylogeny of 43 aeromonads inferred from 1645 core genes by maximum-likelihood using the GTR+Γ model. Only bootstrap values under 100 are shown in this figure. All the bootstrap values for the *salmonicida* subspecies are given on Figure 1 (main article) for clarity. The red branches correspond to mesophilic taxa, the purple branch corresponds to intermediate taxon and the blue branch corresponds to psychrophilic taxa. The strain numbers are shown only when there are two taxa from the same species or subspecies.

**Figure S6.** Average nucleotide identity (ANI) analyses for some *A. salmonicida* subspecies included in this study. *A. bestiarum* is also included for comparative purposes with *A. salmonicida* CBA100. Two taxa were considered as belonging to the same subspecies if they shared an ANI value ≥ 96 (yellow and green).

**Figure S7. Growth curves at 7°C for selected *A. salmonicida* subspecies.** The growth curves were determined three times in independent experiments. The means of three replicates with standard error of the mean are shown for each subspecies.

**Figure S8.** The three high-copy plasmids found in the Indian strain Y47. The pY47-3 plasmid was also found in the Indian strain Y577. The blue arrows represent genes with a known function, the green arrows represent genes encoding hypothetical proteins, and the black arrow represents the putative RNA regulator.

**Figure S9.** The high-copy plasmid pJF4097 found in *A. salmonicida* subsp. *smithia*. The blue arrows represent genes with a known function, the green arrows represent genes encoding hypothetical proteins, the black arrows represent the putative RNAs regulator, and the grey rectangle represents the IS*AS11*.

**Figure S10**. Result of the PCR assay confirming that the RS 534 strain lost its TTSS by the recombination of two IS*AS11*s (B-C rearrangement [10]). The wells are as follows: (1) 2-log DNA ladder (New England Biolabs), (2) RS 534, (3) JF3224 (positive control), and (4) 01-B526 (negative control).

**Figure S11.** Pan-genome analysis of selected *A. salmonicida* subspecies, including *A. popoffii* as an outgroup. (A) Distribution of the pan-genome on a phylogenetic tree for some key taxa. The phylogenetic tree was based on the tree found using the core genome. The green and black values indicate the number of genes acquired and lost, respectively, for the specific branch using the parsimonious Dollo model. The branch lengths represent the number of genes acquired or lost. For *A. salmonicida* subsp. *salmonicida* the strain used was 01-B526. Relative importance of 26 functional categories for the genes implicated in branches 1 (B) and 2 (C).

**Figure S12.** Functional categories of the genes under positive selection in the *A. salmonicida* mesophilic lineages.

**References**

1. Stajich JE, Block D, Boulez K, Brenner SE, Chervitz SA, Dagdigian C, et al. The Bioperl toolkit: Perl modules for the life sciences. Genome Res. 2002;12:1611–8.

2. Altschul SF, Madden TL, Schäffer AA, Zhang J, Zhang Z, Miller W, et al. Gapped BLAST and PSI-BLAST: a new generation of protein database search programs. Nucleic Acids Res. . 1997;25 :3389–402.

3. Seshadri R, Joseph SW, Chopra AK, Sha J, Shaw J, Graf J, et al. Genome sequence of *Aeromonas hydrophila* ATCC 7966T: Jack of all trades. J. Bacteriol. 2006;188:8272–82.

4. Darriba D, Taboada GL, Doallo R, Posada D. jModelTest 2: more models, new heuristics and parallel computing. Nat. Methods. 2012;9:772–772.

5. Jia F, Lo N, Ho SYW. The impact of modelling rate heterogeneity among sites on phylogenetic estimates of intraspecific evolutionary rates and timescales. PLoS One. 2014;9:e95722.

6. Richter M, Rosselló-Móra R. Shifting the genomic gold standard for the prokaryotic species definition. Proc. Natl. Acad. Sci. U. S. A. 2009;106:19126–31.

7. Kurtz S, Phillippy A, Delcher AL, Smoot M, Shumway M, Antonescu C, et al. Versatile and open software for comparing large genomes. Genome Biol. 2004;5:R12.

8. Colston SM, Fullmer MS, Beka L, Lamy B, Gogarten JP. Bioinformatic Genome Comparisons for Taxonomic and Phylogenetic Assignments Using *Aeromonas* as a Test Case. MBio. 2014;5:1–13.

9. Emond-Rheault J-G, Vincent AT, Trudel M V, Frey J, Frenette M, Charette SJ. AsaGEI2b: a new variant of a genomic island identified in the *Aeromonas salmonicida* subsp. *salmonicida* JF3224 strain isolated from a wild fish in Switzerland. FEMS Microbiol. Lett. 2015;362:fnv093.

10. Tanaka KH, Dallaire-Dufresne S, Daher RK, Frenette M, Charette SJ. An Insertion Sequence-Dependent Plasmid Rearrangement in *Aeromonas salmonicida* Causes the Loss of the Type Three Secretion System. PLoS One. 2012;7:e33725.

11. Reith ME, Singh RK, Curtis B, Boyd JM, Bouevitch A, Kimball J, et al. The genome of *Aeromonas salmonicida* subsp. *salmonicida* A449: insights into the evolution of a fish pathogen. BMC Genomics. 2008;9:427.

12. Galardini M, Biondi EG, Bazzicalupo M, Mengoni A. CONTIGuator: a bacterial genomes finishing tool for structural insights on draft genomes. Source Code Biol. Med. 2011;6:11.

13. Rice P, Longden I, Bleasby A. EMBOSS: The European Molecular Biology Open Software Suite. Trends Genet. 2000;16:276–7.

14. Aziz RK, Bartels D, Best AA, DeJongh M, Disz T, Edwards RA, et al. The RAST Server: rapid annotations using subsystems technology. BMC Genomics. 2008;9:75.

15. Shao Y, Harrison EM, Bi D, Tai C, He X, Ou HY, et al. TADB: A web-based resource for Type 2 toxin-antitoxin loci in bacteria and archaea. Nucleic Acids Res. 2011;39:D606–11.

16. Rasko DA, Rosovitz MJ, Økstad OA, Fouts DE, Jiang L, Cer RZ, et al. Complete sequence analysis of novel plasmids from emetic and periodontal *Bacillus cereus* isolates reveals a common evolutionary history among the *B. cereus*-group plasmids, including *Bacillus anthracis* pXO1. J. Bacteriol. 2007;189:52–64.

17. Bolger AM, Lohse M, Usadel B. Trimmomatic: a flexible trimmer for Illumina sequence data. Bioinformatics. 2014;30:2114–20.

18. Liu Y, Popp B, Schmidt B. CUSHAW3: sensitive and accurate base-space and color-space short-read alignment with hybrid seeding. PLoS One. 2014;9:e86869.

19. García-Alcalde F, Okonechnikov K, Carbonell J, Cruz LM, Götz S, Tarazona S, et al. Qualimap: Evaluating next-generation sequencing alignment data. Bioinformatics. 2012;28:2678–9.

20. Pavan ME, Abbott SL, Zorzópulos J, Janda JM. *Aeromonas salmonicida* subsp. *pectinolytica* subsp. nov., a new pectinase- positive subspecies isolated from a heavily polluted river. Int. J. Syst. Evol. Microbiol. 2000;50:1119–24.

21. Abbott SL, Cheung WKW, Janda JM. The genus *Aeromonas*: Biochemical characteristics, atypical reactions, and phenotypic identification schemes. J. Clin. Microbiol. 2003;41:2348–57.

22. Vincent AT, Boyle B, Derome N, Charette SJ. Improvement in the DNA sequencing of genomes bearing long repeated elements. J. Microbiol. Methods. 2014;107:186–8.

23. Valdes N, Espinoza C, Sanhueza L, Gonzalez A, Corsini G, Tello M. Draft Genome Sequence of the Chilean isolate Aeromonas salmonicida strain CBA100. FEMS Microbiol. Lett. 2015;362:fnu062.

24. Pavan ME, Pavan EE, López NI, Levin L, Pettinari MJ. Living in an extremely polluted environment: clues from the genome of melanin-producing *Aeromonas salmonicida* subsp. *pectinolytica* 34melT. Appl. Environ. Microbiol. 2015;81:5235–48.

25. Overbeek R, Olson R, Pusch GD, Olsen GJ, Davis JJ, Disz T, et al. The SEED and the Rapid Annotation of microbial genomes using Subsystems Technology (RAST). Nucleic Acids Res. 2014;42:D206–14.

26. Samson JE, Magadán AH, Sabri M, Moineau S. Revenge of the phages: defeating bacterial defences. Nat. Rev. Microbiol. 2013;11:675–87.

27. Sugiyama T, Itoh T. Control of ColE2 DNA replication: in vitro binding of the antisense RNA to the Rep mRNA. Nucleic Acids Res. 1993;21 :5972–7.

28. Han JE, Kim JH, Choresca JH, Shin SP, Jun JW, Chai JY, et al. First description of ColE-type plasmid in *Aeromonas* spp. carrying quinolone resistance (*qnrS2*) gene. Lett. Appl. Microbiol. 2012;55:290–4.

29. Horii T, Itoh T. Replication of ColE2 and ColE3 plasmids: The regions sufficient for autonomous replication. Mol. Gen. Genet. MGG. 1988;212:225–31.

30. Yahr TL, Vallis AJ, Hancock MK, Barbieri JT, Frank DW. ExoY, an adenylate cyclase secreted by the *Pseudomonas aeruginosa* type III system. Proc. Natl. Acad. Sci. U. S. A. 1998;95:13899–904.

31. Boyd J, Williams J, Curtis B, Kozera C, Singh R, Reith M. Three small, cryptic plasmids from *Aeromonas salmonicida* subsp. *salmonicida* A449. Plasmid. 2003;50:131–44.

32. Spataro N, Farfán M, Albarral V, Sanglas A, Lorén JG, Fusté MC, et al. Draft Genome Sequence of *Aeromonas molluscorum* Strain 848TT, Isolated from Bivalve Molluscs. Genome Announc. 2013;1:e00382–13.

33. Charette SJ, Brochu F, Boyle B, Filion G, Tanaka KH, Derome N. Draft genome sequence of the virulent strain 01-B526 of the fish pathogen *Aeromonas salmonicida* . J. Bacteriol. 2012;194:722–3.

34. Vincent AT, Tanaka KH, Trudel M V, Frenette M, Derome N, Charette SJ. Draft genome sequences of two *Aeromonas salmonicida* subsp. *salmonicida* isolates harboring plasmids conferring antibiotic resistance. FEMS Microbiol. Lett. 2015;362:1–4.

35. Han JE, Kim JH, Shin SP, Jun JW, Chai JY, Park SC. Draft Genome Sequence of *Aeromonas salmonicida* subsp. *achromogenes* AS03, an Atypical Strain Isolated from Crucian Carp (*Carassius carassius*) in the Republic of Korea. Genome Announc. 2013;1:e00791–13.

36. Pavan ME, Pavan EE, López NI, Levin L, Pettinari MJ. Genome Sequence of the Melanin-Producing Extremophile *Aeromonas salmonicida* subsp. *pectinolytica* Strain 34melT. Genome Announc. 2013;1:e00675–13.

37. Gurevich A, Saveliev V, Vyahhi N, Tesler G. QUAST: quality assessment tool for genome assemblies. Bioinformatics. 2013;29:1072–5.
